# Divergent regulation of long non-coding RNAs *H19* and *PURPL* affects cell senescence in human dermal fibroblasts

**DOI:** 10.1007/s11357-024-01399-3

**Published:** 2024-10-22

**Authors:** Elena Frediani, Cecilia Anceschi, Jessica Ruzzolini, Sara Ristori, Alice Nerini, Anna Laurenzana, Anastasia Chillà, Claudia Elena Zoe Germiniani, Gabriella Fibbi, Mario Del Rosso, Alessandra Mocali, Marco Venturin, Cristina Battaglia, Lisa Giovannelli, Francesca Margheri

**Affiliations:** 1 Department of Experimental and Clinical Biomedical Sciences, University of Florence, Viale G.B. Morgagni, 50 – 50134 Florence, Italy; 2 Department of Neurofarba (Department of Neurosciences, Drug Research and Child Health), University of Florence, Viale Pieraccini, 6 – 50139 Florence, Italy; 3 Department of Medical Biotechnology and Translational Medicine, University of Milan, Via Vanvitelli, 32 – 20133 Milan, Italy

**Keywords:** Cell senescence, Long non-coding RNAs, H19, PURPL, Human fibroblasts

## Abstract

Cellular senescence is a permanent cell growth arrest that occurs in response to various intrinsic and extrinsic stimuli and is associated with cellular and molecular changes. Long non-coding RNAs (lncRNAs) are key regulators of cellular senescence by affecting the expression of many important genes involved in senescence-associated pathways and processes. Here, we evaluated a panel of lncRNAs associated with senescence for their differential expression between young and senescent human dermal fibroblasts (NHDFs) and studied the effect of a known senomorphic compound, resveratrol, on the expression of lncRNAs in senescent NHDFs. As markers of senescence, we evaluated cell growth, senescence-associated (SA)-β-Gal staining, and the expression of p21, Lamin B1 and γH2AX. We found that *H19* and *PURPL* were the most altered lncRNAs in replicative, in doxorubicin (DOXO) and ionising radiation (IR)-induced senescence models. We then investigated the function of *H19* and *PURPL* in cell senescence by siRNA-mediated silencing in young and senescent fibroblasts, respectively. Our results showed that *H19* knockdown reduced cell viability and induced cell senescence and autophagy of NHDFs through the regulation of the PI3K/AKT/mTOR pathway; conversely, *PURPL* silencing reversed senescence by reducing (SA)-β-Gal staining, recovering cell proliferation with an increase of S-phase cells, and reducing the p53-dependent DNA damage response. Overall, our data highlighted the role of *H19* and *PURPL* in the senescent phenotype and suggested that these lncRNAs may have important implications in senescence-related diseases.

**Supplementary Information:**

The online version contains supplementary material available at 10.1007/s11357-024-01399-3.

## Introduction

Cellular senescence is a state of permanent arrest of cell growth. In 1965, Hayflick defined the limited lifespan of primary fibroblasts as cellular senescence [1]. Subsequent studies have shown that this process in fibroblasts is known as replicative senescence, a particular type of senescence caused by telomere erosion. Cell senescence can also be triggered by a variety of factors other than replication stress, without corresponding shortening of telomeres, including DNA damage, oxidative stress, oncogenic induction, or proteasome inhibition, the so-called stress-induced premature senescence (SIPS) [2]. Senescent cells exhibit characteristic molecular and morphological features that make them distinguishable from normal cells [3]. The expression of lysosomal hydrolase activity at pH 6.0, senescence-associated β-galactosidase (SA-β-Gal), and the accumulation of cytoplasmic granules in the lysosomes leading to an enlarged hypertrophic and flattened morphology are characteristic phenotypes of senescent cells [4, 5]. At the molecular level, the expression of *p16*^*INK4a*^ and p21^*CIP1*^, cyclin-dependent kinase inhibitors, that induce the cell cycle arrest in G1, is a prominent marker of cellular senescence. In addition, an emerging hallmark of senescence is the reduction of lamin B1 expression [6, 7], which reflects the disruption of the nuclear lamina, a typical feature of senescent cells. Although senescent cells are growth arrested in the cell cycle, they are still metabolically active [8]. These cells secrete a wide spectrum of bioactive factors, including inflammatory cytokines, chemokines, growth factors, matrix metalloproteinases, lipids, nucleotides, extracellular vesicles, and soluble factors, termed the senescence-associated secretory phenotype (SASP) [9]. The release of DNA from the nucleus, cytoplasmic chromatin fragments (CCFs), and NF-κB pathway activation play a crucial role in priming the SASP [10, 11].

Recent studies have revealed the contribution of different classes of non-coding RNAs to cellular senescence. Long noncoding RNAs (lncRNAs) are an emerging large and heterogeneous group of non-protein-coding transcripts longer than 200 nucleotides, which participate in several biological processes, including transcription, post-transcription, and epigenetic regulation of gene expression [12, 13] and are involved in a variety of cellular functions related to cellular senescence, proliferation, differentiation, and the cell response to stress or immunogens [14]. LncRNAs show functional plasticity, such as signalling, molecular bait, molecular guidance, and scaffold formation [15]. In particular, lncRNAs are able to regulate gene expression in different ways, including acting as bait or sponges for microRNAs (miRNAs) [16–18]. Several studies have indicated that lncRNAs play crucial regulatory roles in the process of senescence [19, 20]. Expression of lncRNAs has been altered during senescence. Moreover, a number of lncRNAs can induce or deactivate cellular senescence [14]. Despite the rapidly rising interest in the expression and function of lncRNAs, their possible implication in senescence remains virtually unexplored.

On this basis, this study aimed to screen for lncRNAs that might be related to the senescence process and capable of modulating the senescent phenotype. We evaluated a panel of lncRNAs previously associated with senescence for their differential expression between young (proliferating) and senescent human dermal fibroblasts (NHDFs). Among the senescence-associated lncRNAs, we selected two, H19 and *PURPL* (also called *LINC01021*), as candidate lncRNAs and evaluated their function in modulating of the senescent phenotype using three strategies: (1) assessing the expression of *H19* and *PURPL* expression in NHDF-induced senescence; (2) assessing of the effect of a known senomorphic compound, resveratrol, on the expression of *H19* and *PURPL* in senescent fibroblasts; (3) assessing of the effect of siRNA-mediated silencing of *H19* and *PURPL*, in young and senescent fibroblasts respectively, on NHDF-induced senescence.

## Methods

### Cell cultures

Neonatal human dermal fibroblasts (NHDFs) were obtained from Lonza (Euroclone, Pero, Italy). NHDFs were cultured in high-glucose (4500 g/L) Dulbecco’s Modified Eagle Medium (DMEM), supplemented with 10% foetal bovine serum, 2 mM L-glutamine, 100 units/mL penicillin, and 100 µg/mL streptomycin (Sigma-Aldrich, Milan, Italy) at 37 °C in a humidified atmosphere of 5% CO_2_. At confluence, cultures were propagated by trypsinization, and the attained population doubling level (PDL) was calculated for NHDFs according to the equation: PDL = 3.32 × log 10 (*N*/*N*0) (where *N* and *N*0 are the recovered and seeded cell numbers, respectively).

### Replicative senescence and resveratrol treatment

The experiments with NHDF fibroblasts were conducted on young, proliferating (PDL < 20), pre-senescent (PDL 20–35), and senescent (PDL > 35) cultures, and after 5 weeks of propagation in culture with or without 5 μM final resveratrol (Sigma-Aldrich) concentration, these cells became senescent as described previously [20–22] (see also Supporting information).

### Doxorubicin-induced senescence

Before starting the experiments, sufficient pre-senescent NHDFs were seeded for 24 h in a complete medium at 37 °C in a 5% CO_2_ humidified incubator. Subsequently, fibroblasts were treated with 50 nM Doxo for 48 h to induce senescence and then cultured in a complete medium for 3 days as previously described [23] (see also Supporting information).

### Ionising radiation (IR)-induced senescence

Pre-senescent NHDF cultures were seeded, grown to confluence, and then irradiated with γ-radiation delivered at 146 Gy/h with a 137Cs source using a GammaCell 1000 irradiator as described previously [24]. Single doses of 6, 8, and 10 Gy were delivered to the cell cultures. Pre-senescent cells mock-irradiated (placed inside the irradiator for the same interval used for the irradiated samples, but without exposure to radiation) were used as a control.

### RNA isolation

Total RNA was extracted from NHDF using TRIZOL. The concentration and purity of RNA were measured using the Nanodrop 1000 spectrophotometer (ThermoFisher Scientific). All RNA samples had an A260/280 value of 1.8–2.1. The quality of RNA was also evaluated using a Tape Station 2200 instrument (Agilent). All the samples had a RIN value ≥ 9. One microgram of total RNA was treated with the RQ1 RNase-Free Dnase (M6101, Promega), and then, cDNA was synthesised in 20 µl reactions using the High-Capacity cDNA Reverse Transcription Kit (4,368,814, Applied Biosystems), according to the manufacturer’s instructions.

### Quantitative real-time PCR for lncRNA expression

Quantitative RT-PCR was performed with the QuantStudio 5 thermocycler (Applied Biosystems) in 384-well plates using the GoTaq qPCR Master Mix (A6002, Promega). Ten microliters PCR reactions was prepared containing 2 µl of reverse transcriptase product and 0.2 µl of each primer (10 µM) for specific genes (Table 1). The PCR mixtures were initially denatured at 95 °C for 2 min, followed by 40 cycles of 95 °C for 10 s and 60 °C for 1 m. The results were analysed using the QuantStudio Design & Analysis Software v1.5.2 (Applied Biosystems). The melting curve showed a single product peak, indicating good product specificity. The calculation of gene expression levels was based on the 2^-(ΔΔCt) method using the geometric mean of the expression values of three normaliser genes (*CYC1*, *EIF4A2*, and *RPSA*). Fold changes were calculated using 2-(ΔΔCt) and compared between senescence/treated versus young/untreated NHDF cells.

**Table 1:** Primers used for q-RT-PCR experiments (protein-coding genes and lncRNAs)

Gene	FWD	RVS
CDKN1A (p21)	5′-TGGAGACTCTCAGGGTCGAAA-3′	5′-GGCGTTTGGAGTGGTAGAAATC -3′
CDKN2A (p16)	5′-CATAGATGCCGCGGAAGGT-3′	5′-TCTAAGTTTCCCGAGGTTTCTCA-3′
CXCL8 (IL8)	5′-ATACTCCAAACCTTTCCACCC-3′	5′-TCTGCACCCAGTTTCCTTG-3′
EIF4A2	5′-GGTCAGGGTCAAGTCGTGTT-3′	5′-CCCCCTCTGCCAATTCTGTG-3′
GAS5	5′-CTCAAGCCATTGGCACACAGG-3′	5′-CGTTACCAGGAGCAGAACCA-3′
GLB1	5′-AATCAAGACCGAAGCAGTGGC-3′	5′-GGGTGAGTTGGCCCCATTCC-3′
H19	5′-ACGGAGTCGGCACACTATGG-3′	5′-CAGGCTTGAGCTGGGTAGCA-3′
HMGB1	5′-AGAAGCCGAGAGGCAAAATGT-3′	5′-ATAACGGGCCTTGTCCGCTT-3′
HOTAIR	5′-GGCAAGACGGGCACTCACAG-3′	5′-CTGGGCGTTCATGTGGCGAG-3′
IL1b	5′-CCAGGGACAGGATATGGAGCA-3′	5′-TTCAACACGCAGGACAGGTACAG-3′
IL6	5′-CCACTCACCTCTTCAGAACG-3′	5′-CATCTTTGGAAGGTTCAGGTTG-3′
LMNB1	5′-GAACCAGAACTCGTGGGGCA-3′	5′-ATGCTCTTGGGGTTCCCTGC-3′
MEG3	5′-TCCCACCCCTCTTGCTTGTC-3′	5′-CCATCCGCAGTTCTTCAGCC-3′
NEAT1	5′-CGGAGGTGAGGGGTGGTCTG-3′	5′-GCAGTCCCCGCCTGTCAAAC-3′
PURPL	5′-ATCCGGCTATTTTGGAGATTGA-3′	5′-AGCCTGGACCACAGAACAAG-3′
RPSA	5′-CAACAACAAGGGAGCTCACTC-3′	5′-CTTCTCAGCAGCAGCCTGCT-3′
TP53	5′-GGCCCCTCCTCAGCATCTTATC-3′	5′-GGCACAAACACGCACCTCAA-3′
TUG1	5′-AGCATCTCACAAGGCTGCAC-3′	5′-CAAAGGGCTTCATGGCCACA-3′

### (SA)-β-Gal assay

After induction of senescence using different models, for the assessment of senescence-associated (SA)-β-Gal activity, cells were seeded at a density of 10–20 cells cm^2^. (SA)-β-Gal-staining was performed according to the method described by Dimri et al. 1995 [25] (see also Supporting information).

### Western blot analysis

Thirty to forty micrograms of lysate proteins for each sample was used for Western Blot analyses together with the molecular weight Magic Mark (Invitrogen, Carlsbad, CA, USA). The list of antibodies and working conditions is given in Supplementary Table S1 (see Supporting Information for details).

### Confocal immunofluorescence

Confocal immunofluorescence analysis of γ-H2Ax, NFkB, and p53 was performed as previously described [24] (see Supporting information for details). Total NFkB fluorescence intensity and Mander’s coefficient (M1), used to assess NFκB p65 colocalization with the nucleus (DAPI), were determined using ImageJ software.

### Knockdown of H19 and PURPL by small interfering RNAs

Targeting and not-targeting siRNAs were obtained from Dharmacon (Carlo Erba Reagents, Milan, Italy) and Life Technologies Italia (Monza, Italy). Specific silencing of *H19* was performed by transfection of young NHDFs and silencing of *PURPL* was performed by transfection of senescent NHDFs, with small interfering RNA, according to the manufacturer’s instructions (see Supporting Information for details).

### Flow cytometry analysis of the cell cycle

NHDF cultures were seeded in a six-well plate at a density of 1.5 × $1{0}^{5}$ cells/well in DMEM supplemented with 10% FBS. Cells were starved for 48 h in DMEM 0.2% FBS. After 48 h, cells were incubated in DMEM with FBS 10% for 24 h. Cells were harvested with 500 μl of propidium iodide solution and incubated for 30 min at 4 °C. Cell cycle was analysed on a BDLSR cytofluorimeter (BD Biosciences) using FlowJo software (BD Bioscience).

### Statistical analysis

Unless otherwise stated, all experiments were performed three times in duplicate for a reliable statistical application. Statistical analysis was performed using GraphPad Prism 9.0 software. The values are expressed as the mean ± standard deviation (SD). An unpaired two-tailed Student’s *t*-test was performed for comparison of *n* = 2 groups. Comparisons of *n* > 2 groups were performed using one-way ANOVA along with Bonferroni’s post hoc test. For all statistical tests, *p* ≤ 0.05 was considered statistically significant and ≤ 0.01 very statistically significant.

## Results

### Characterisation of replicative senescence in NHDFs and analysis of senescence-associated lncRNAs

**Fig. 1:**
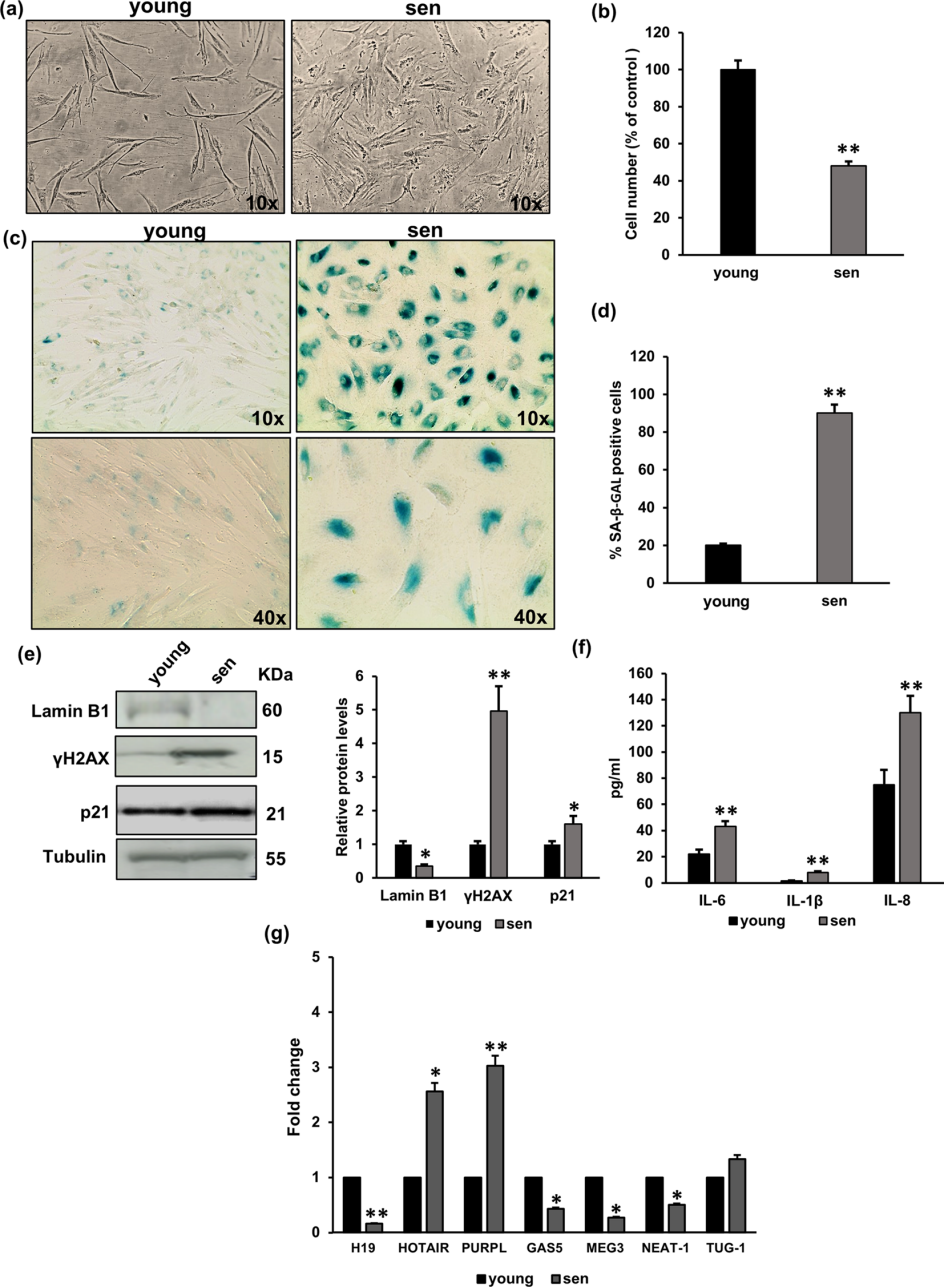
Replicative senescence markers and characterisation of senescence-associated lncRNAs. **a** NHDFs morphology either at low (young) or high (sen) PDL; images are representative of cell cultures photographed using a phase contrast microscope (× 200 final magnification). **b** Histograms report the senescent cells number compared to young fibroblasts. **p < 0.01 vs. young NHDFs. **c** Representative images of (SA)‐β‐Gal‐positive fibroblasts are reported (× 100 magnification). **d** Percentage of (SA)‐β‐Gal‐positive cells. **p < 0.01 vs. young NHDFs. **e** Western blotting analysis of LaminB1, γH2AX, and p21 in young and in senescent fibroblasts. Protein bands were normalised to α-tubulin. Histograms reported γH2AX, LaminB1, and p21 protein quantification. *p < 0.05 vs young NHDFs. **p < 0.01 vs NHDFs. **f** IL-1B, IL-6, and IL-8 levels are measured in conditioned media by ELISA assay. The histograms report the pg/ml of the selected cytokines. **p < 0.01 vs NHDFs. **g** Histograms report fold gene expression of senescence-associated lncRNAs, comparing senescence versus young NHDF cells. *p < 0.05 vs untreated NHDFs. **p < 0.01 vs untreated NHDFs. In all the graphs, bars are the mean (± *SD*) of three experiments

To analyse the expression of senescence-related lncRNAs, we exploited a model of replicative senescence of NHDFs. Therefore, NHDF fibroblasts, either at low (young) or high (sen) PDL, were analysed for typical markers of cellular senescence and related to SASP. Figure 1a and b show that sen NHDFs have an enlarged and flattened morphology and a reduced cell number (55%) compared to young fibroblasts. In parallel, the expression of the typical senescence marker, SA-βGal is higher in sen NHDFs with an increase in the percentage of positive cells (45%) compared with young NHDFs (Fig. 1c and d). Figure 1e reports the expression levels of, LaminB1, γH2AX, and p21 in young and sen NHDFs by western blotting. Sen NHDFs showed an increase in p21(WAF1/Cip1), a factor that promotes cell cycle arrest. Moreover, we observed in senescent fibroblast an increased expression of the phosphorylated form of the histone variant H2AX, a marker of DNA damage, and a reduced level of Lamin B1, a major constituent of nuclear lamina, indicating nuclear DNA leakage associated with cellular senescence. Considering SASP-related factors, the levels of all the main cytokines, IL-1B, IL-6, and IL-8, were significantly increased in conditioned media collected from senescent fibroblasts compared with those collected from young fibroblasts (Fig. 1f). Once the senescent phenotype of NHDFs was characterised, we then analysed the expression of several lncRNAs that might be related to the senescence process. A panel of 7 lncRNAs was manually selected from the current literature according to their association with senescence (Table 2). Among the 7 selected lncRNAs (Table 2), the gene expression analysis revealed a significant upregulation of *PURPL* and *HOTAIR* and a downregulation of *H19* and *MEG3* in senescent cells compared with young NHDFs (Fig. 1g). In particular, the lncRNAs *PURPL* and *H19* were the most decreased and increased respectively, in senescent fibroblasts.

**Table 2:** List of lncRNAs selected for this study and their role in senescence

lncRNA symbol	lncRNA name	Expression in cellular senescence	Function/mechanism of action	References
H19	H19 imprinted maternally expressed transcript	Up/down	Inhibits p53, CDKN1C, IL-6/STAT3/p21 pathway (anti-senescence function). Derepresses β-catenin (pro-senescence function)	Puvvula (2019)
HOTAIR	HOX transcript antisense RNA	Up	Activates NF-κB/IL-6 and p53/p21 pathways through DNA damage response	Puvvula (2019)
GAS5	Growth arrest-specific 5	--	Sponges miR-223 which inhibits the anti-senescence NAMPT enzyme	Cao et al. (2020)
MEG3	Maternally expressed 3	Up	Enhances p53 transcription and reduces p53 degradation. Promotes p53 binding to target promoters	Grammatikakis et al. (2014); Puvvula (2019)
NEAT1	Nuclear paraspeckle assembly transcript 1	--	Facilitates the expression of IL-8	Cao et al. (2020)
PURPL	p53 upregulated regulator of p53 levels	Up	Negatively controls p53 levels by interfering with p53-MYBBP1A complex	Li et al. (2017); Casella et al. (2019)
TUG1	Taurine up-regulated 1	Up	Growth arrestor induced by p53 upon DNA damage. Inhibits the pro-proliferation HOXB7 transcription factor	He et al. (2018)

### H19 and PURPL are associated with stress-induced premature senescence (SIPS) of NHDFs

**Fig. 2:**
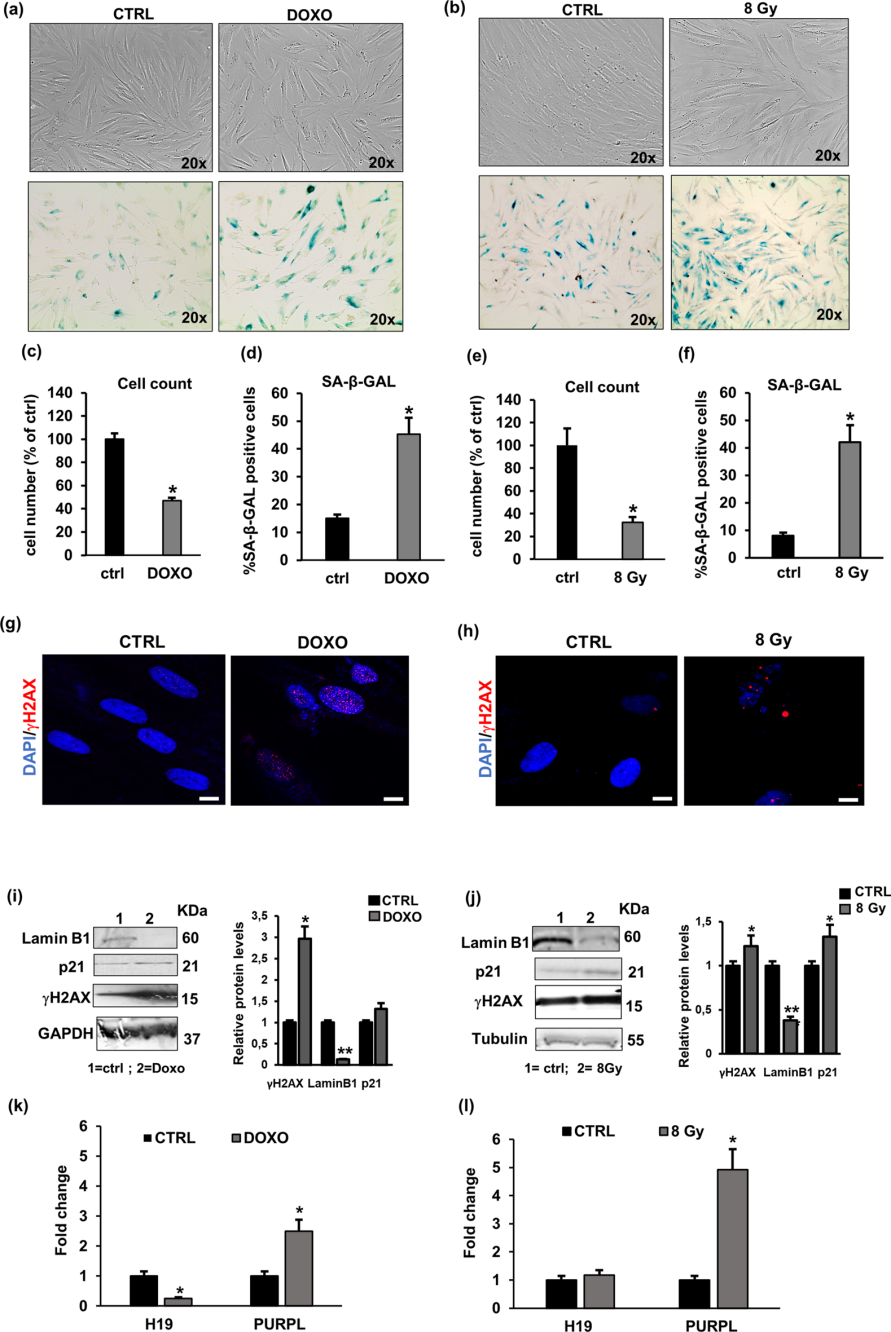
Characterisation of stress-induced premature senescence (SIPS) and SIPS-associated lncRNAs. **a** Pre-senescent NHDFs were treated with Doxo 50 nM for 48 h and then cultured in fresh complete medium for the following 72 h; images are representative of NHDFs photographed using a phase contrast microscope (× 100 magnification). Representative images of SA-β-Gal activity performed after DOXO treatment. **b** Confluent pre‐senescent NHDF cultures were exposed to doses of ionising radiation (8 Gy) and mock irradiation was used as a control (CTRL). Representative images of cell cultures photographed after 8 Gy irradiation using a phase contrast microscope (× 200 final magnification). Representative images of (SA)‐β‐Gal‐positive cells after 8 Gy treatment are reported. **c** Histograms report the percentage cell number of Doxo 50 nM treated NHDFs compared to untreated fibroblasts (CTRL). *p < 0.05 vs untreated NHDFs. **d** Percentage of (SA)‐β‐Gal‐positive Doxo treated cells. *p < 0.05 vs untreated NHDFs. **e** Histograms report the percentage cell number of 8 Gy-treated NHDFs vs untreated fibroblasts (CTRL). * *p* < 0.05 vs mock-irradiated cells (CTRL). **f** Percentage of (SA)‐β‐Gal‐positive of 8 Gy-treated cells. *p < 0.05 vs mock‐irradiated cells (CTRL). **g** Confocal analysis of γH2AX expression in NHDFs after Doxo treatment. **h** Confocal analysis of γH2AX expression in NHDFs after 8 Gy treatment. **i** Western blotting analysis of LaminB1, γH2AX, and p21 in untreated fibroblasts (CTRL) and fibroblasts treated with Doxo 50 nM; protein bands were normalised to GAPDH. Histograms report γH2AX, LaminB1and p21 protein quantification. *p < 0.05 vs untreated NHDFs. **p < 0.01 vs untreated NHDFs. **j** Western blotting analysis of LaminB1, γH2AX, and p21 in untreated NHDFs (CTRL) and 8 Gy-treated NHDFs; protein bands were normalised to GAPDH. Histograms report γH2AX, LaminB1, and p21 protein quantification. *p < 0.05 vs. untreated NHDFs. **p < 0.01 vs untreated NHDFs. **k** Relative expression of lncRNAs H19 and PURPL comparing Doxo-treated vs untreated NHDFs. *p < 0.05 vs untreated NHDFs. (l) Fold change gene expression of lncRNAs *H19* and *PURPL* comparing 8 Gy-treated vs mock-irradiated cells (CTRL). In all the graphs, bars are the mean (± *SD*) of three experiments

To further confirm the role of *H19* and *PURPL* in the senescence process triggered by different stimuli, we analysed their expression in models of stress-induced premature senescence (SIPS). In particular, we induced cell senescence in dermal fibroblasts by treatment with doxorubicin, a chemotherapeutic agent, and by ionising radiation (IR) exposure, as previously described [24]. As reported in Fig. 2a and b, fibroblasts treated with 50 nM doxorubicin for 48 h (DOXO) or irradiated with 8 Gy dose (8 Gy) acquired the typical morphology of senescent cells, with a larger and flatter body than CTRL cells that exhibit an elongated shape, and the number of recovered cells was significantly lower in DOXO and 8 Gy-treated cells (53% and 68% reduction, respectively) compared to CTRL NHDFs (Fig. 2c and e). Similarly, the percentage of SA-βGal positive cells was increased up to 45% in both treatments with respect to CTRL (Fig. 2a, b and d, f). Moreover, Fig. 2g and h show that the number of γH2AX positive nuclear foci was increased after DOXO or IR exposure compared with CTRL. These data were also confirmed by western blotting analysis of γH2AX. (Fig. 2i and j) As shown in Fig. 2i and j, p21 protein levels increased, indicating cell growth arrest, whereas lamin B1 was reduced in DOXO and 8 Gy-treated NHDFs compared with CTRL NHDFs. Regardless of the different stimuli able to induce a senescence program, we evaluated levels of *H19* and *PURPL* by real-time PCR. As reported in Fig. 2k and l, after treatment with DOXO or 8 Gy dose, *PURPL* was upregulated, while *H19* was downregulated, only after DOXO exposure compared with CTRL.

### H19 and PURPL are modulated by resveratrol treatment in replicative NHDF senescence

**Fig. 3:**
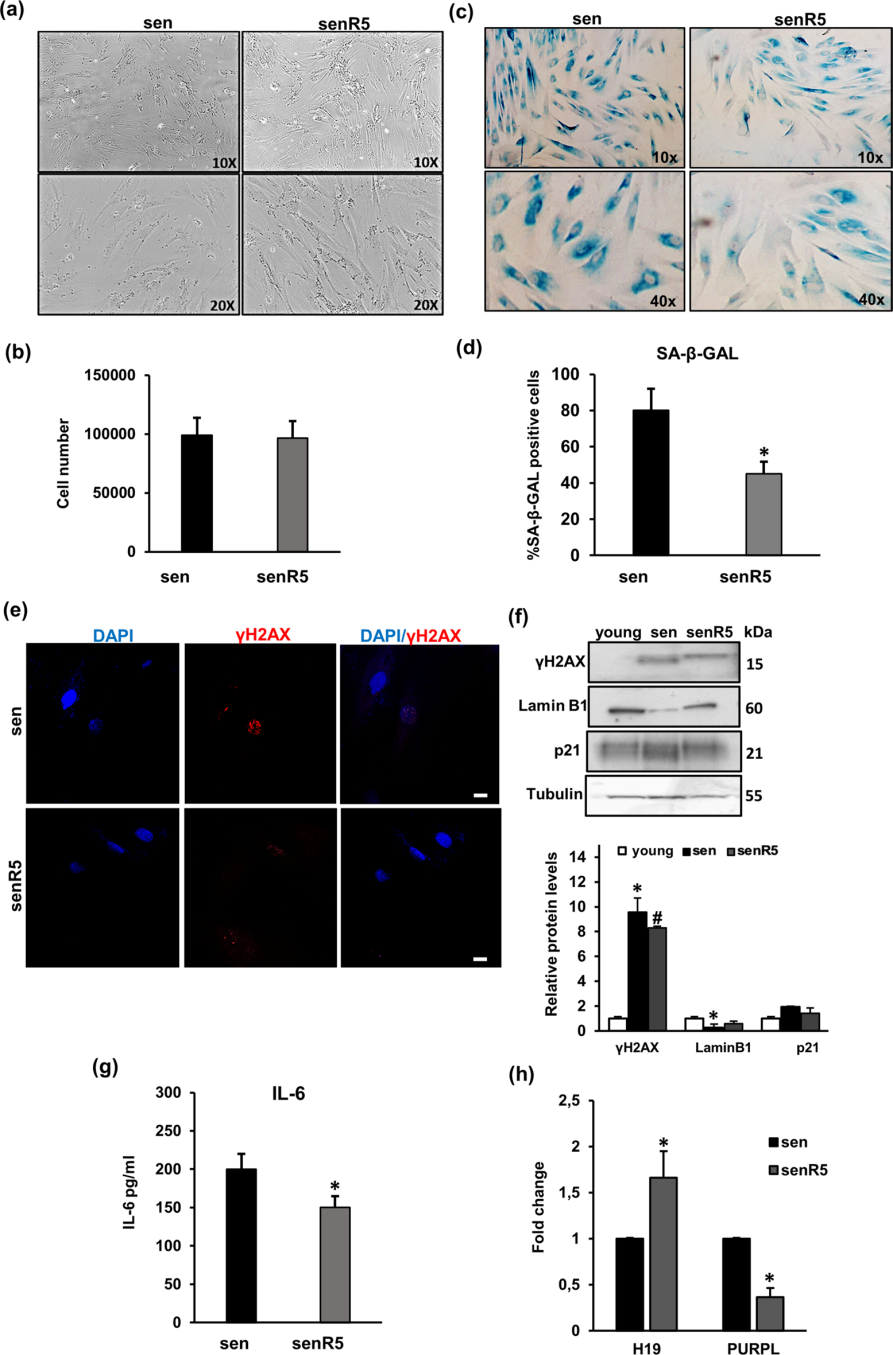
Modulatory effect of resveratrol on replicative senescence. **a** Pre-senescent NHDFs were subjected to chronic treatment with resveratrol 5 µM for 5 weeks, reaching PDL > 35 (senR5); images are representative of cell cultures photographed using a phase contrast microscope (× 100 magnification). **b** Histograms report cell number of resveratrol 5 µM treated NHDFs (senR5) compared to senescent fibroblasts (CTRL). **c** Representative images of (SA)‐β‐Gal‐positive cells are reported (× 200 final magnification). **d** Percentage of (SA)‐β‐Gal‐positive cells. *p < 0.05 vs untreated NHDFs. **e** Confocal analysis of γH2AX expression in NHDFs R5. Scale bar = 10 µm. **f** Western blotting analysis of LaminB1, γH2AX, and p21 in young, sen, and senR5 fibroblasts; protein bands were normalised to tubulin. Histograms report γH2AX, LaminB1, and p21 protein quantification. *p < 0.05 vs young NHDFs, #*p* < 0.05 vs sen NHDFs. **g** Histograms report pg/ml of IL-6. *p < 0.05 vs untreated NHDFs. **h** Histograms report fold change gene expression of *H19* and *PURPL*, comparing R5-treated versus untreated NHDFs. *p < 0.05 vs untreated NHDFs. In all the graphs, bars are the mean (± *SD*) of three experiments

To further investigate the role of *H19* and *PURPL* expression and the senescent phenotype, we used a pharmacologic approach that exploited the senescence modulatory effect of resveratrol, a natural stilbenoid phenol with well-characterised senotherapeutic properties in vitro [22, 26] and in vivo. As for the in vivo effects of resveratrol, starting with the work of D. Sinclair [27], it has been repeatedly shown that the long-term treatment with this molecule can delay age-related deterioration in animal models of different age-related pathologies, such as in a model of aortic calcification [28], in one of ethanol-induced pancreas senescence [29], and in aged fish [30]. In our model in vitro, pre-senescent NHDFs were subjected to chronic treatment with resveratrol 5 µM for 5 weeks (R5) as previously described [22, 26]. Figure 3a shows that resveratrol (R5), as expected, is able to attenuate the morphological alterations of senescent cells, although it does not cause any change in proliferation activity compared with senescent fibroblasts (sen). In fact, the number of cells recovered from resveratrol cultures after 5 weeks of treatment was not significantly different from that of sen NHDFs (Fig. 3b). As reported in Fig. 3c and d, R5 determined a reduction in the percentage of SA-β-Gal positive cells with respect to senescent NHDFs (sen). Similarly, resveratrol treatment induced a reduction in several senescence markers such as γH2AX, as evident by confocal immunofluorescence analysis in sen and senR5 NHDFs (Fig. 3e) and western blotting (Fig. 3f), and lamin B1 (Fig. 3f). No significant changes were observed in the p21 protein levels (Fig. 3f). Moreover, we have used IL-6 release as a marker of the whole SASP [31, 32], to confirm the ability of resveratrol to reduce SASP, and we found that R5 treatment resulted in a small but significant reduction of IL-6 released in conditioned media from R5-treated compared with control NHDFs (sen) (from 200 pg/ml to 167 ng/ml) (Fig. 3g). Figure 3h shows the expression of senescence-associated lncRNAs by real-time PCR. Among them, R5 treatment was able to significantly affect the expression of *H19* and *PURPL*. In particular, *H19*, which was downregulated in senescent fibroblasts, was increased by R5 treatment, and conversely, *PURPL*, which was upregulated lncRNA senescent cells, was reduced in R5-treated compared with sen NHDFs.

### H19 knockdown reduced cell viability and promoted NHDF cell senescence

**Fig. 4:**
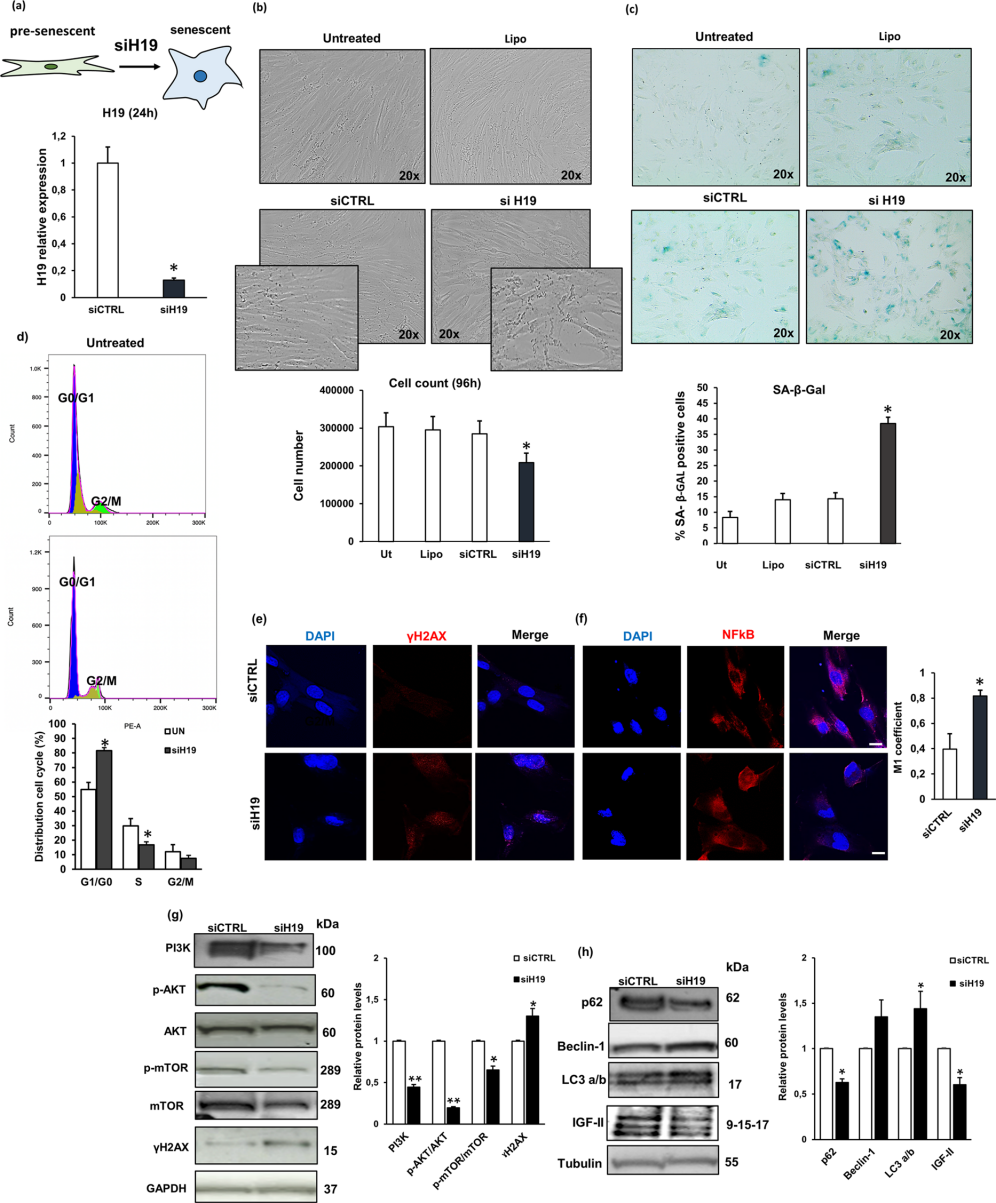
Effect of *H19* silencing on cell viability and on the induction of cellular senescence. **a** *H19* knockdown was performed by transient transfection of young NHDFs with small-interfering-RNA (siRNA). Histograms report fold change gene expression of *H19* in siH19 versus siCTRL NHDFs. Bars are the mean (± *SD*) of three experiments. *p < 0.05 vs siCTRL NHDFs analysed by *t*-test. **b** Representative images of cell morphology after *H19* silencing were photographed using a phase contrast microscope (on the top). Histograms report the percentage of cell number. Bars are the mean (± *SD*) of three experiments. *p < 0.05 vs siCTRL NHDFs. **c** Representative images of (SA)‐β‐Gal‐positive cells (× 200 final magnification) (on the top). Percentage of (SA)‐β‐Gal‐positive cells after H19 silencing. Bars are the mean (± *SD*) of three experiments. *p < 0.05 vs ctrl NHDFs. **d** Cell cycle analysis in siH19 and untreated NHDFs using the FlowJo software (BD Bioscience). Histograms report the percentage of cell cycle distribution in siH19 compared to untreated NHDFs. Bars are the mean (± *SD*) of three experiments. *p < 0.05 and **p < 0.01 vs untreated NHDFs. **e**, **f** Confocal analysis of γH2AX and NFkB expression in siH19 and siCTRL NHDFs. Scale bar = 10 µm. Histogram shows the quantification of NFkB nuclear localization (NFkB/DAPI) by Mander’s coefficient (M1) using ImageJ software. **g**, **h** Western blotting analysis of PI3K, p-AKT, AKT, p-mTOR, mTOR, γH2AX, p62, Beclin-1, LC3, and IGF-2 in siH19 and siCTRL NHDFs; GAPDH and Tubulin were used as loading control. Histograms report PI3K, p-AKT/AKT, p-mTOR/mTOR, γH2AX, p62, Beclin-1, LC3, and IGF-2 protein quantification. *p < 0.05 vs siCTRL NHDFs. **p < 0.01 vs siCTRL NHDFs. In all the graphs, bars are the mean (± *SD*) of three experiments

Next, to further evaluate the impact of *H19* on cell viability and in the induction of cellular senescence of NHDFs, we performed loss-of-function experiments with specific siRNA transfected into young NHDFs. As shown in Fig. 4a, H19 expression was remarkably downregulated in siH19 compared with siCTRL NHDFs, confirming the efficacy of siRNA-mediated silencing (87% reduction with respect to siCTRL). As indicated in Fig. 4b, H19 silencing determined the acquisition of enlarged and flattened morphology and a 30% reduction of cell number recovered 96 h, after transfection, compared with untreated, non-targeting, and LIPO-transfected NHDFs. SA-β-Gal assay showed that H19 knockdown significantly enhanced the percentage of positive cells compared with ctrl NHDFs (untreated, siCTRL and LIPO) (Fig. 4c). To explore the effect of *H19* silencing on cell proliferation, we analysed the cell cycle progression in siCTRL and siH19 NHDFs by flow cytometry. Figure 4d shows that the percentage of cells in the S phase of the cell cycle is lower (16% vs 29.9%) in siH19 than in siCTRL cells. Moreover, we have observed in siH19-transfected cells an increase of γH2AX expression by western blotting (Fig. 4g) and confocal immunofluorescence (Fig. 4e). At the molecular level, we analysed the activation of the PI3K/AKT/mTOR pathway involved in cell viability [33] and autophagy [34], and of NFkB, the master regulator of SASP phenotype. As reported in Fig. 4g, the phosphorylation of PI3K, AKT, and mTOR was significantly reduced after *H19* downregulation. At the same time, IGF-2, a molecular target of *H19*, significantly decreased in siH19 compared to siCTRL NHDFs (Fig. 4f). Considering the markers of autophagic flux, Beclin-1 and LC3 results increased, while p62 slightly reduced, after *H19* silencing (Fig. 4f). Furthermore, the nuclear translocation of NFkB was increased by *H19* silencing, as evident by confocal analysis of the M1 coefficient (50% increase of M1 value in siH19 compared with siCTRL NHDFs) (Fig. 4f). Taken together, *H19* silencing reduced NHDF cell proliferation and facilitated cell senescence and autophagy.

### PURPL knockdown enhanced cell viability and dampened cell senescence in NHDFs through p53 regulation

**Fig. 5:**
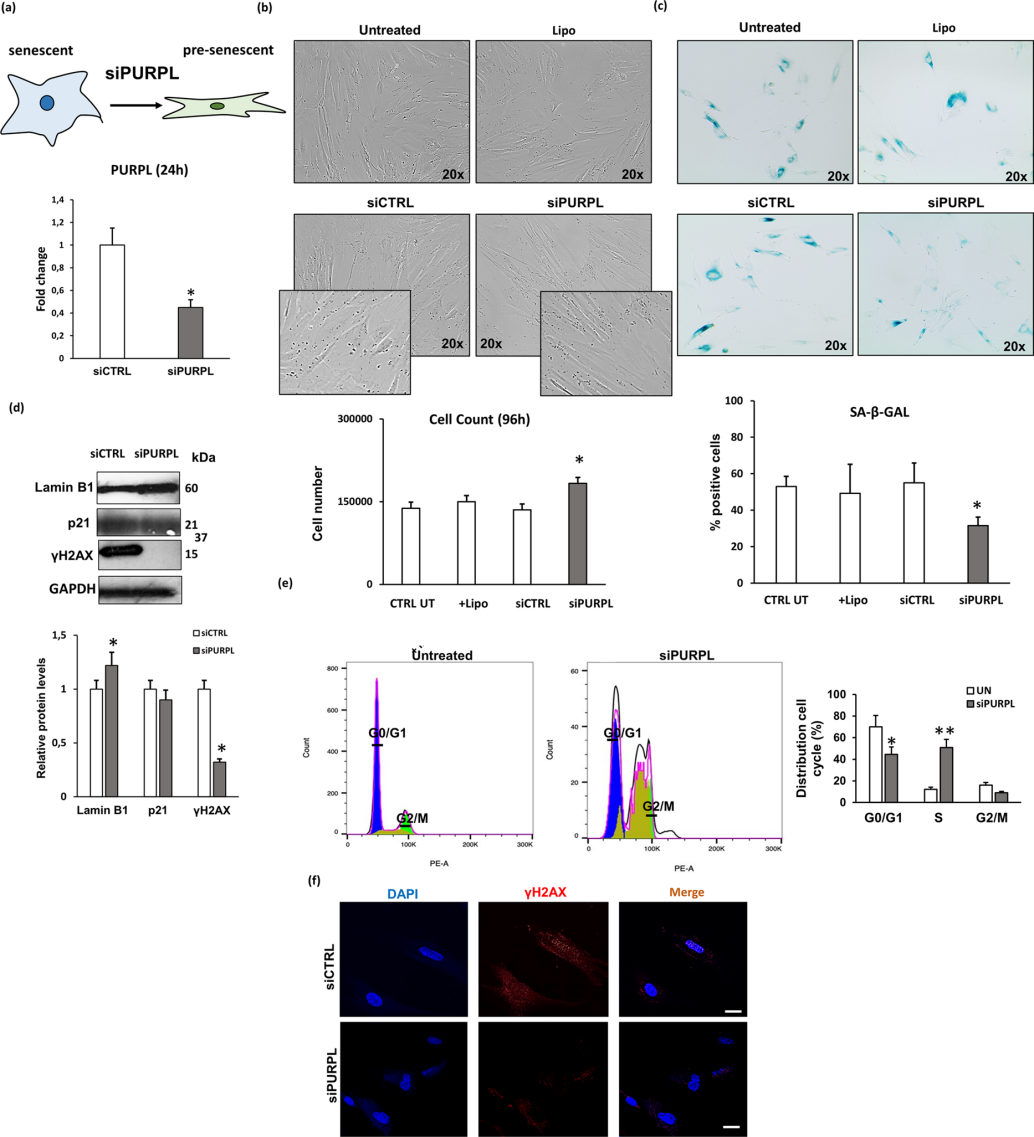
Effect of *PURPL* silencing on cell viability and on the induction of cellular senescence. **a** *PURPL* knockdown was performed by transient transfection of senescent NHDFs with small-interfering-RNA (siRNA). Histograms report fold change gene expression of *PURPL* in siPURPL versus siCTRL NHDFs. Bars are the mean (± *SD*) of three experiments. *p < 0.05 vs siCTRL NHDFs analysed by *t*-test. **b** Representative images of NHDFs after *PURPL* silencing by phase contrast microscope (× 200 final magnification). Histograms report the percentage of cell number in siPURPL-transfected compared to ctrl NHDFs (untreated, siCTRL, and LIPO). Bars are the mean (± *SD*) of three experiments. *p < 0.05 vs siCTRL NHDFs. **c** Representative images of (SA)‐β‐Gal‐positive cells (on the bottom). Percentage of (SA)‐β‐Gal‐positive cells. Bars are the mean (± *SD*) of three experiments. *p < 0.05 vs siCTRL NHDFs. **d** Western blotting analysis of LaminB1, γH2AX, and p21 in siPURPL and siCTRL NHDFs; GAPDH was used as a loading control. Histograms report γH2AX, LaminB1, and p21 protein quantification. *p < 0.05 vs siCTRL NHDFs. **e** Cell cycle analysis in siPURPL and siCTRL NHDFs using the FlowJo software (BD Bioscience). Histograms report the percentage of cell cycle distribution in siPURPL compared to untreated NHDFs. Bars are the mean (± *SD*) of three experiments. *p < 0.05 and ** *p* < 0.01 vs untreated NHDFs. **f** Confocal analysis of γH2AX expression in siPURPL and siCTRL NHDFs. Scale bar = 10 µm. In all the graphs, bars are the mean (± *SD*) of three experiments

With regard to the function of *PURPL* on cell viability and senescence status of NHDFs, we performed loss-of-function experiments with specific siRNA transfected into senescent cells. Similarly, to *H19* silencing, once the sub-confluence condition (80%) was reached, senescent NHDFs (PDL > 35) were transiently transfected with targeting (siPURPL) and not-targeting (siCTRL) siRNAs and with the transfection reagent only (LIPO). Figure 5a reports the results of real-time PCR of *PURPL* performed 24 h after transfection. *PURPL* expression was downregulated in siPURPL compared with siCTRL NHDFs, without reaching the levels achieved by silencing *H19* (55% versus 87%). As evident in Fig. 5b, after *PURPL* silencing, senescent cells exhibited evident changes in cell morphology with a more regular and elongated shape, similar to young cells, compared with siCTRL cells. Moreover, cell viability slightly but significantly increased (35%) in siPURPL cells, with a higher number of cells recovered 96 h after transfection compared with siCTRL cells. As indicated in Fig. 5c, the expression of SA-β-Gal was strongly reduced in fibroblasts transfected with siPURPL compared with siCTRL. Similarly, we have observed in siPURPL-transfected cells a reduction of γH2AX expression by western blotting (Fig. 5d) by confocal immunofluorescence (Fig. 5f) and an increase in lamin B1 expression. No differences were observed in p21 levels. To further investigate the effect of *PURPL* silencing on cell proliferation, we analysed cell cycle progression in siCTRL and siPURPL NHDFs by flow cytometry. Figure 5e shows that the percentage of cells in the S phase of the cell cycle was higher (50% vs 12%) in siPURPL than in untreated (UN) cells that accumulated in the G1 phase (more than 70%).

**Fig. 6:**
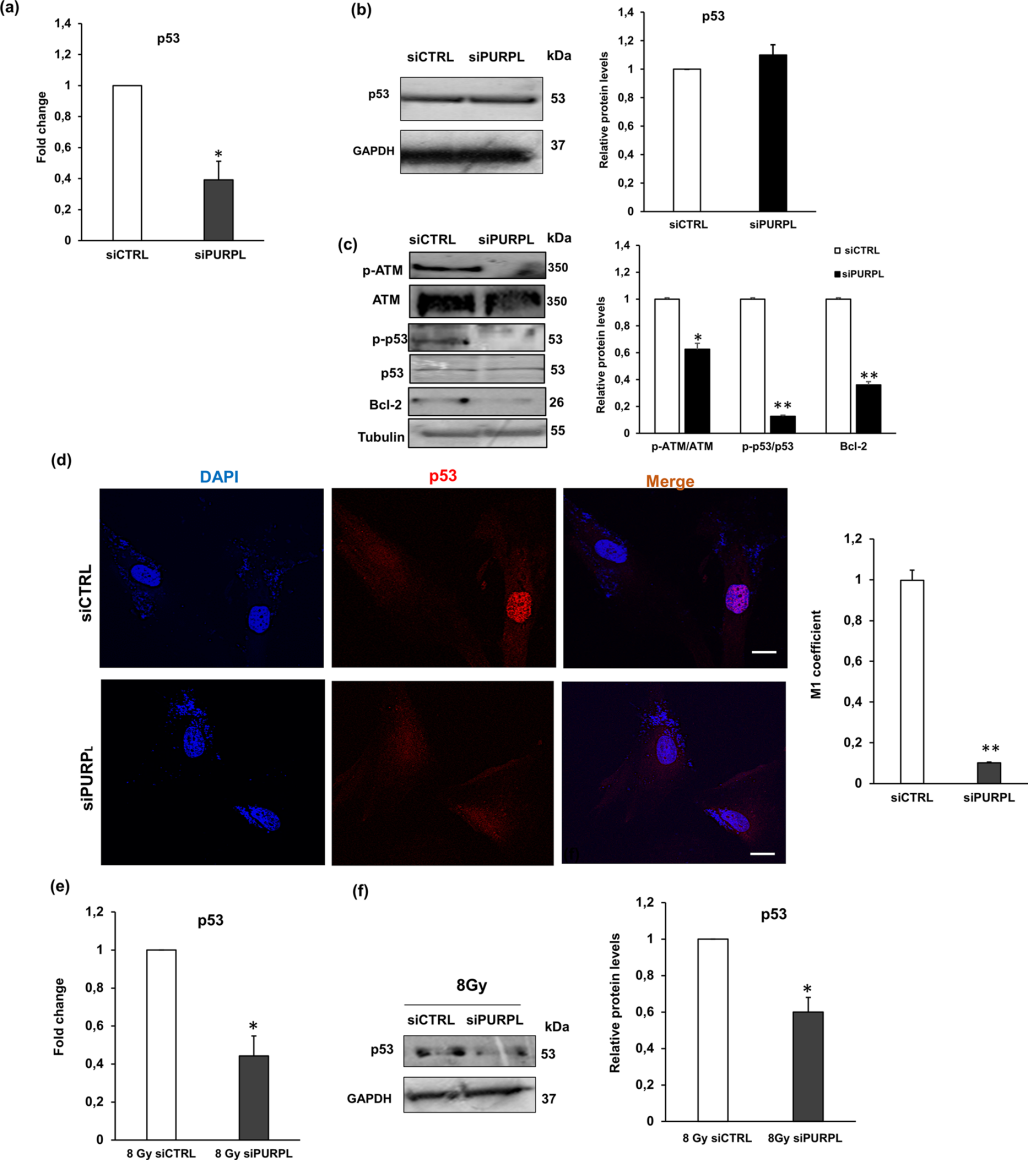
Effect of *PURPL* knockdown on p53 expression. **a** p53 fold change gene expression in siPURPL and siCTRL NHDFs. *p < 0.05 vs siCTRL NHDFs. **b** Western blotting analysis of p53 in siPURPL and siCTRL NHDFs; GAPDH was used as a loading control. Histograms report p53 protein quantification. *p < 0.05 vs siCTRL. **c** Western blotting analysis of p-ATM, ATM, p-p53, p53, and Bcl-2 in siPURPL and siCTRL NHDFs; Tubulin was used as loading control. Histograms report p-ATM/ATM, p-p53/p53, and Bcl-2 protein quantification. *p < 0.05 vs siCTRL NHDFs. **p < 0.01 vs siCTRL NHDFs. **d** Confocal analysis of p53 in siPURPL and siCTRL NHDFs. Scale bar = 10 µm. **e** Histogram reports the quantification of p53 nuclear localization (NFkB/DAPI) by Mander’s coefficient (M1) using ImageJ software. **f** left panel: p53 fold change gene expression in siPURPL and siCTRL 8 Gy NHDFs. *p < 0.05 vs siCTRL (8 Gy NHDFs). **f** right panel: Western blotting analysis of p53 in siPURPL and siCTRL NHDFs; GAPDH was used as a loading control. Histograms report p53 protein quantification. *p < 0.05 vs 8 Gy siCTRL. In all the graphs, bars are the mean (± *SD*) of three experiments

Next, we explored whether *PURPL* silencing could impact the level of endogenous p53 in NHDFs. First, we analysed the mRNA and protein levels of p53 in siCTRL and siPURPL NHDFs. Figure 6a shows a reduction of p53 at the mRNA level in siPURPL compared with siCTRL NHDFs, while no difference was observed at the protein level (Fig. 6b). At the same time, we investigated the activation of ATM, p53, and Bcl-2. As reported in Fig. 6b, we observed a reduction of ATM and p53 phosphorylation, as well as a decrease of Bcl-2 (Fig. 6b). Then, to evaluate p53 nuclear translocation, we performed confocal immunofluorescence analysis in siCTRL and siPURPL NHDFs. As shown in Fig. 6c and d, we observed a more nuclear positivity in siCTRL than in siPURPL NHDFs. Moreover, to further investigate how *PURPL* modulation could affect p53 levels, we analysed p53 expression in IR-exposed NHDFs after *PURPL* silencing. We observed that *PURPL* downregulation in IR-exposed fibroblasts resulted in a significant reduction of p53 at the mRNA and protein level (Fig. 6e and f).

## Discussion

Increasing evidence suggests that the expression of lncRNAs plays a key role in several biological processes, as well as in the induction and maintenance of cellular senescence. In this study, we have analysed the expression of senescence-related lncRNAs in a replicative NHDF senescence model. Senescent NHDFs showed a reduction of cell growth and exhibited both the typical enlarged morphology and the expression of SA-β-Gal, as well as increased γH2AX and p21, and reduced Lamin B1. In the conditioned media of sen NHDFs, we detected an increase in released SASP-related factors (IL-6, IL-1β, and IL-8) compared with young NHDFs. Concerning lncRNA expression, *HOTAIR* and *PURPL* were overexpressed, whereas *H19*, *NEAT1*, and *MEG3* were downregulated in senescent compared with young fibroblasts. *H19* and *PURPL* were the most increased and decreased respectively in replicative senescence and we confirmed this data also in stress-induced premature senescence (SIPS), by treatment with doxorubicin and by ionising radiation. Moreover, the long-term treatment of replicative senescent NHDFs with resveratrol (5 µM for 5 weeks, R5), a well-known senomorphic compound, was able to upregulate *H19* and simultaneously reduce *PURPL* expression. As *H19* and *PURPL* were the most increased and decreased respectively, among the analysed lncRNAs, we decided to perform siRNA-mediated knockdown of *H19* and *PURPL* in pre-senescent and senescent NHDFs, respectively. *H19* silencing was effective in inducing premature senescence by assessing three main parameters: the total number of cells collected and counted at 96-h post-transfection, (SA)‐β‐Gal activity, and DNA damage evaluation by γH2AX expression. In parallel, in siH19 NHDFs, we observed activation of NF_k_B, the master regulator of the senescent and SASP phenotype. Furthermore, we found a significant reduction of AKT and mTOR phosphorylation after H19 silencing, indicating a role of *H19* in the regulation of the AKT/mTOR axis and, subsequently, in cell proliferation and autophagy. In particular, concerning the autophagic flux, we have observed an increase in Beclin-1 and LC3, markers of autophagosomes, and a slight reduction in p62 after *H19* knockdown.

Our data about *H19* are consistent with previous observations suggesting a role for this lncRNA in counteracting cellular senescence. *H19*, first identified as an imprinting lncRNA, is overexpressed at the foetal stage and downregulated postpartum, except in the skeletal muscle system [35]. The *H19* level has been documented to correlate with the development of age-related disorders by modulating inflammation and providing protection against age-related diseases [36]. In addition, it was previously discovered that *H19* upregulation facilitates wound healing of skin tissue [37]. This lncRNA is under-expressed in the endothelial cells of aged mice. Similarly, the expression of *H19* was found to be down-regulated in atherosclerotic plaques compared with normal carotid arteries [38]. Regarding H19 expression in human dermal fibroblasts, Tang et al. have highlighted an “anti-senescent” effect of *H19* on dermal fibroblast by impairing microRNA-296-5p-dependent inhibition of IGF2 [33]. Similarly, we observed a downregulation of this lncRNA in a doxorubicin-induced senescence model but not in IR-induced senescence. The reason for this difference might lie in the complexity of the *H19* function. In fact, some papers have described an association between *H19* and DNA damage/repair. However, the type of response can vary dramatically depending on cell type, probably due to the different miRNAs regulated [39–42]. No data is available on fibroblasts. In the models of induced senescence that we have used, we have observed a very high DNA damage in the DOXO model and a more modest increase in the irradiation one. Thus, we can hypothesise that the differential degree of DNA damage attained in these two senescence models is associated with different degrees of *H19* expression inhibition.

Regarding *PURPL*, we have, for the first time, validated the role of this lncRNA in fibroblast senescence by performing a loss-of-function experiment using specific siRNAs in senescent NHDFs. We observed a lower efficiency compared with *H19* silencing, reaching a reduction of *PURPL* expression of approximately 65% versus 80% for *H19*. A possible explanation for this result could be the functional differences between senescent and young NHDFs used for transfection. Despite this lower transfection efficiency, after *PURPL* silencing, we observed an improvement of cell morphology with a more regular and elongated shape and a reduction of SA-β-GAL and γH2AX positive cells. In parallel, we found a significant recovery in cell proliferation activity, as evident from the number of cells collected and counted at 96-h post-transfection and from cell cycle analysis indicating a higher percentage of cells in the S phase compared with CTRL cells. These data are particularly interesting considering that we performed knockdown in senescent fibroblasts and, surprisingly, we obtained a gain in cell proliferation. However, we carried out *PURPL* silencing experiments in NHDF at intermediate and not late senescence status.

*PURPL* (P53 upregulated regulator of P53 levels) is an intergenic lncRNA also termed *LINC01021*. It has been discovered to modulate p53 to boost liver cancer cell proliferation and differentiation [43]. Another study showed that lncRNA *PURPL* and miR-338-3p dysregulation can be adopted as biomarkers of epithelial ovarian cancer prognosis [44]. Similarly, *PURPL* regulates p53 levels in colorectal cancer (CRC) cell lines [45]. Although it was first identified in 2019 as one of the most strikingly elevated transcripts in various senescent cell models (replicative cell exhaustion, DOXO, or IR exposure) in WI-38 and IMR-90 fibroblasts and in human umbilical vein endothelial cells (HUVECs) [46], the function of *PURPL* in fibroblast senescence has never been validated. Here, we confirmed in senescent NHDFs a significant upregulation of *PURPL* in replicative and stress-induced senescence models according to these previous observations. In particular, we have shown that *PURPL* is also modulated in two different stress-induced premature senescence models, one using doxorubicin, a chemotherapeutic agent that induces a DNA damage response in cells leading to activation of the p53 pathway, and the other using IR-induced senescence.

Equally noteworthy are the data concerning p53 expression. First, we analysed the expression of p53 and *PURPL* in cell senescence. Our data showed that *PURPL* is upregulated in different models of cell senescence and that *PURPL* and p53 expression are co-dependent. We then explored whether *PURPL* silencing could affect the level of endogenous p53 in NHDFs. After *PURPL* knockdown, we found a significant reduction in p53 mRNA levels, but no difference was observed at the total protein level. However, analysing the confocal images of p53, we observed a more nuclear positivity of p53 in siPURPL than in siCTRL fibroblasts. These findings suggest that *PURPL* silencing did not affect p53 protein levels or transcriptional activity in NHDFs, but modified the subcellular distribution of p53, reducing the nuclear levels of the protein. Conversely, in IR-induced senescence, we showed that *PURPL* knockdown affects p53 at the mRNA and protein levels. At the same time, we observed a reduction of ATM and p53 phosphorylation, as well as a decrease of Bcl-2 in siPURPL compared to siCTRL fibroblasts, indicating that *PURPL* affects the DNA damage sensing and response pathways and the apoptosis resistance.

To date, the relationship between p53 and *PURPL* expression in fibroblasts has not been investigated. p53 is a master tumour suppressor, considered the ‘guardian of the genome’, as it is the most frequently mutated gene in human cancers [47–49]. It plays key roles in cell cycle control, apoptosis, senescence, DNA repair, and changes in metabolism and metastasis through the transcriptional regulation of its target genes [50]. Numerous findings and conflicting results are related to p53-*PURPL* interactions in cancer cells. A recent study on hepatocellular carcinoma (HCC) showed that the lncRNA *PURPL* is upregulated in HCC biopsies and that its expression is p53-dependent in liver cancer cell lines [51]. Another study reported that loss of *PURPL* sensitises ColoRectal Cancer (CRC) cells towards the chemotherapeutic drugs DOXO and 5-FU, suggesting that *PURPL* is involved in the p53-mediated response to DNA damage. However, modulating *PURPL* expression did not affect p53 protein levels or transcriptional activity [52]. In another study, *PURPL* was shown to suppress basal p53 levels and promote CRC tumourigenicity, where MYBBP1A was identified as a *PURPL*-interacting protein through RNA pull-down studies [45]. *PURPL* is known to be a p53 target, because its promoter contains p53-response elements. In colorectal cancer cells, *PURPL* is transcriptionally activated by p53 and, in return, it can decrease the levels of p53 and its targets, such as p21. Mechanistically, *PURPL* can negatively regulate p53 stability by inhibiting its interaction with the MYBBP1A protein, which can bind and stabilise p53. These discrepant results in HCC and CRC illustrate the complexity of *PURPL*-p53 interactions, and further investigations are needed.

In summary, our study provides novel insight into the role of *H19* and *PURPL* in cellular senescence and suggests that these lncRNAs could have pathogenic roles in senescence-related disorders. Identification of new biomarkers and molecular targets, such as *H19* and *PURPL*, and subsequent characterisation of the cellular mechanisms are urgently needed to develop new therapeutic strategies for cellular senescence. Manipulation of the senescence process has been suggested as a therapeutic option for several disorders. While pro-senescent treatments are desirable therapeutic options in malignant conditions and for tissue repair processes, anti-senescent strategies can be helpful for the removal of senescent cells in the contexts of ageing, age-associated diseases, or chronic injuries [53]. Induction of cellular senescence can enhance the response of cancer cells to therapeutic options. Identification of the impact of non-coding RNAs on the regulation of cellular senescence has practical significance in cancer treatment. Moreover, because abnormal expression of non-coding RNAs in cancer cells can have anti-senescence effects, evaluation of the levels of these transcripts in tumour samples can facilitate the prior evaluation of the response of tumour cells to chemoradiotherapy. This field represents an unexplored research area and opens the way for the design of novel therapies for a wide variety of human disorders, including ageing-related disorders and cancers.

## Supplementary Information

Below is the link to the electronic supplementary material.

Supplementary file1 (DOCX 26 KB)

## Data Availability

The datasets used and/or analysed during the current study are available from the corresponding author on reasonable request.

## Abbreviations

SIPS: Stress-induced premature senescence
SA-β-Gal: Senescence-associated β-galactosidase
SASP: Senescence-associated secretory phenotype
LncRNA: Long noncoding RNA
miRNAs: MicroRNAs
H19: H19 imprinted maternally expressed transcript
PURPL: P53 upregulated regulator of P53 levels
NHDFs: Neonatal human dermal fibroblasts
PDL: Population doubling level
DOXO: Doxorubicin
IR: Ionising radiation
R5: Resveratrol 5 µM for 5 weeks
siRNA: Small interfering RNA
qRT-PCR: Quantitative real-time PCR
