# Supplementary material for: Divergent regulation of long non-coding RNAs H19 and PURPL affects cell senescence in human dermal fibroblasts
Source: GeroScience. 2024 Oct 22;47(2):2079–97. doi: 10.1007/s11357-024-01399-3 (PMC11979041; doi:10.1007/s11357-024-01399-3)
Supplement: Supplementary file 1 — Supplementary file1 (DOCX 26 KB) [file 11357_2024_1399_MOESM1_ESM.docx]

**Divergent regulation of long non-coding RNAs *H19* and *PURPL* affects cell senescence in human dermal fibroblasts.**

**Elena Frediani^1^, Cecilia Anceschi^1^, Jessica Ruzzolini^1^, Sara Ristori^1^, Alice Nerini^2^, Anna Laurenzana^1^, Anastasia Chillà^1^, Claudia Elena Zoe Germiniani^3^, Gabriella Fibbi ^1^, Mario Del Rosso^1^, Alessandra Mocali^1^, Marco Venturin^3,^ Cristina Battaglia^3^, Lisa Giovannelli^2#^, and Francesca Margheri^1#^.**

**^1^Department of Experimental and Clinical Biomedical Sciences, University of Florence, Viale G.B. Morgagni, 50 – 50134, Florence, Italy**

**^2^Department of Neurofarba (Department of Neurosciences, Psychology, Drug Research and Child Health), University of Florence, Viale Pieraccini, 6 – 50139, Florence, Italy**

**Corresponding authors: Francesca Margheri:** [**francesca.margheri@unifi.it**](mailto:francesca.margheri@unifi.it) **,**

**Lisa Giovannelli:** [**lisa.giovannelli@unifi.it**](mailto:lisa.giovannelli@unifi.it)

**Supplementary material**

**Supplementary table S1**

| **Table S1: Antibodies used for Western blotting** |  |  |  |
| --- | --- | --- | --- |
| **PRIMARY ANTIBODY** | **COMPANY-CATALOG NUMBER** | **RRID** | **DILUTION** |
| **GAPDH (14C10)** | **Cell Signaling-2118** | AB_561053 | 1:1000 |
| **Phospho-histone H2AX (Ser139) (20E3)** | **Cell Signaling-9718** | AB_2118009 | 1:1000 |
| **LaminB1 (D9V6H)** | **Cell Signaling-13435** | AB_2737428 | 1:1000 |
| **p21 Waf1/Cip1 (12D1)** | **Cell Signaling-2947** | AB_823586 | 1:1000 |
| **p53 (DOI-1)** | **Santa Cruz- sc-126** | AB_628082 | 1:500 |
| **Phosho-mTOR (Ser2448)** | **Cell Signaling-5536** | AB_10691552 | 1:1000 |
| **mTOR** | **Cell Signaling-2972** | AB_330978 | 1:1000 |
| **Phospho-Akt (Ser473)** | **Cell Signaling-9271** | AB_329825 | 1:1000 |
| **Akt** | **Cell Signaling-9272** | AB_329827 | 1:1000 |
| **Anti-α-Tubulin AntibodyDM1A, monoclonal** | **Sigma-Aldric- T6199** | AB_477583 | 1:1000 |
| **NFKB p65** | **Cell signaling-8242** | AB_10859369 | 1:1000 |
| **PI3 Kinase** | **Cell signaling-4249** | AB_2165248 | 1:1000 |
| **SQSTM1/p62** | **Cell signaling** -**88588** | AB_2800125 | 1:1000 |
| **LC3A/B** | **Cell signaling-4108** | **AB_2137703** | 1:1000 |
| **IGF-II (E3F3N)** | **Cell signaling-40941** | **NA** | 1:1000 |
| **p-ATM (Ser1981)** | **Cell signaling-4526** | AB_2062663 | 1:1000 |
| **ATM (D2E2)** | **Cell signaling-2873** | AB_2062659 | 1:1000 |
| **p-p53 (Ser15)** | **Cell signaling-9286** | AB_331741 | 1:1000 |
| **Bcl-2** | **Cell signaling-15071** | AB_2744528 | 1:1000 |
| **Beclin-1 (D40C5)** | **Cell signaling-3495** | AB_1903911 | 1:1000 |
| **SECONDARY ANTIBODY** |  |  |  |
| **Anti-Mouse IgG (Fc specific)–Peroxidase antibody produced in goat** | **Sigma-A0168** | AB_257867 | 1:5000 |
| **Anti-Rabbit IgG (Fc specific)-Peroxidase antibody produced in goat** | **Sigma-A 0545** | AB_257896 | 1:5000 |
|  |  |  |  |

**RRID**: Research Resource Identifiers

**Supplementary Materials and Methods**

**Replicative senescence and resveratrol treatment**

The experiments with NHDF fibroblasts were conducted on young, proliferating (PDL< 20), pre-senescent (PDL >20<35) and senescent (PDL>35) cultures, and after 5 weeks of propagation in culture with or without 5 μM final resveratrol (Sigma-Aldrich) concentration, these cells became senescent. At the end of treatment, senescent control and resveratrol-treated NHDF cultures were referred to as sen and senR5, respectively. Low PDL (<30) NHDF proliferating fibroblasts were also propagated in culture and referred to as young. During treatment, control and R5-treated fibroblasts were analyzed for their proliferative activity by viable (Trypan-blue negative) cell counting; in parallel, senescence-associated β-galactosidase staining and p21 protein levels were determined at t = 0 and at t = 30 days.

**Doxorubicin-induced senescence**

Before starting the experiments, sufficient pre-senescent NHDFs were seeded for 24 h in complete medium at 37°C in a 5% CO_2_ humidified incubator. Subsequently, fibroblasts were treated with 50nM Doxo for 48h to induce senescence, and then cultured in complete medium for 3 days. The control was represented by cells incubated for 48 h in complete medium with 0.0005% DMSO (used as diluent for Doxo) and then maintained in fresh complete medium for the following 3 days. At this time, senescence markers were analyzed.

**(SA)-β-Gal Assay**

After induction of senescence using different models, for the assessment of senescence-associated (SA)-β-Gal activity, cells were seeded at a density of 10–20 cells cm^2^. (SA)-β-Gal-staining was performed according to the method described by Dimri *et al.,* 1995 [25]. Cells were washed once with phosphate-buffered saline (PBS), fixed with 3.7% formaldehyde, and rinsed three times with water. Thereafter, cells were incubated with staining buffer [1 mg/ml−1 X-Gal (5-bromo-4-chloro-3-indolyl β-d-galactoside), 5 mM K3Fe [CN]6,5 mM K4Fe [CN]6, 150 mM NaCl and 2 mM MgCl2 in PBS, pH 6.0] for 18 h at 37°C, rinsed with water and air-dried. The local blue precipitate formed by the cleavage of the substrate X-Gal at pH 6.0 in senescent fibroblasts was assessed under phase-contrast microscopy at ×200 magnification and the positive cells were counted.

**Western blot analysis**

Thirty-forty micrograms of lysate proteins for each sample together with the molecular weight Magic Mark (Invitrogen, Carlsbad, CA, USA) were subjected to 4–12% sodium dodecyl sulfate–polyacrylamide gel electrophoresis separation (Bis-Tris Plus BOLT, Invitrogen) and transferred to polyvinylidene fluoride membranes (PVDF, Millipore, Burlington, MA, USA). The membranes were blocked in 5% skim milk and incubated overnight with specific primary antibodies followed by the suitable HRP-conjugated secondary antibodies. All antibodies used are reported in Supplementary Table 1 (Table S1). All resulting immune complexes were visualized using an enhanced chemiluminescence ECL detection system (GE Healthcare, Milano, Italy) and quantified by ImageJ software (NIH, Bethesda, MD, USA).

**Confocal immunofluorescence**

The primary antibodies used in immunofluorescence were rabbit anti-γ-H2Ax (Cell Signaling), rabbit anti-NFkB (p65) (Cell Signaling), and rabbit anti-p53 (7F5) (Cell Signaling), whereas the secondary were Cy3-conjugated goat anti-rabbit or mouse IgG (Sigma-Aldrich Chemicals). DAPI (Sigma-Aldrich Chemicals) was used for nucleus staining. Coverslips with the immune-labeled cells were mounted with an antifade mounting medium (Biomeda, Collegno, Italy) and analyzed under a Bio-Rad MRC 1024 ES confocal laser scanning microscope (Bio-Rad) equipped with a 15-mW Krypton/Argon laser source. Cells were observed with a Nikon Plan Apo X60 oil immersion objective (Nikon Instruments, Rome, Italy) at 595 nm. A series of optical sections (X- and Y-steps: 512 × 512 pixels) were then obtained through the depth of the cells, with a thickness of 1 μm at intervals of 0.8 μm (Z-step). A single composite image was obtained from superimposition of 20 optical sections for each sample. Total NFkB fluorescence intensity and Mander's coefficient (M1), used to assess NFκB p65 colocalization with the nucleus (DAPI), were determined using ImageJ software.

**Knockdown of *H19* and *PURPL* by small interfering RNAs**

Targeting and not-targeting siRNAs were obtained from Dharmacon (Carlo Erba Reagents, Milan, Italy) and Life Technologies Italia (Monza, Italy). Specific silencing of *H19* and *PURPL* was performed by transfection of young (proliferating) and senescent NHDFs respectively, with Silencer select siRNA and with siGENOMESMARTpool siRNA, according to the manufacturer’s instructions. Subconfluent (80%) proliferating or senescent cultures were transfected with 50 nM targeting siRNAs. Not-targeting siRNA pool constructs were used as negative controls (siCONTROL). To promote cell internalization siRNAs were incorporated into cationic liposomes, using 30 nM Lipofectamine 3000 transfection reagent. Cells were incubated with transfection mix (24-48 hr for mRNA analysis and 72-96 hr for protein and phenotypic analysis, respectively).

The silencing of *H19* and *PURPL* with Silencer select siRNAs was performed in order to confirm the data obtained with siGENOMESMARTpool siRNA. In particular, with this second set of siRNA, we analyzed (SA)-β-Gal positivity, γH2AX expression and cell cycle analysis and, for *PURPL* silencing, also Lamin B1 and p21 expression (see Supplementary Figures S1 and S2).

**Supplementary figure legends**

**Figure S1: Effect of *H19* silencing regulation on cell viability and on the induction of cellular senescence (using a second set of siRNA from Life Technologies)**

(a) *H19* knockdown was performed by transfection of young NHDFs with small-interfering-RNA (siRNA). Histograms report relative expression of *H19* comparing siH19-transfected cells vs. siCTRL NHDFs. **p* < 0.05 vs. siCTRL NHDFs. (b) Images are representative of NHDFs after *H19* transfection photographed using a phase contrast microscope (200x final magnification). Histograms report percentage cells number of siH19-transfected cells compared to ctrl NHDFs (untreated, siCTRL). (c) Representative images of (SA)‐β‐Gal‐positive siH19-transfected cells compared to ctrl NHDFs. Percentage of (SA)‐β‐Gal‐positive siH19-transfected cells compared to ctrl NHDFs. **p* < 0.05 vs. ctrl NHDFs. (d) Western blotting analysis of γH2AX in siH19 and siCTRL NHDFs; protein bands were normalized to tubulin. (e) Confocal analysis of γH2AX expression in siH19-transfected NHDFs. Scale bar = 10 μm. (f) Cell cycle analysis in siH19 and siCTRL NHDFs using the FlowJo software (BD Bioscience). Histograms report the percentage of cell cycle distribution in siH19-transfected cells compared to siCTRL NHDFs. In all the graphs, bars are the mean (± *SD*) of three experiments.

**Figure S2: Effect of *PURPL* silencing on cell viability and on the induction of cellular senescence (using a second set of siRNA from Life Technologies)**

(a) *PURPL* knockdown was performed by transfection of senescent NHDFs with small-interfering-RNA (siRNA). Histograms report relative expression of P*URPL* comparing siPUPRL-transfected cells versus siCTRL NHDFs. **p* < 0.05 vs. siCTRL NHDFs. (b) Images are representative of NHDFs after *PURPL* transfection photographed using a phase contrast microscope (200x final magnification). Histograms report percentage cell number of siPURPL-transfected cells compared to ctrl NHDFs (untreated, siCTRL). (c) Representative images of (SA)‐β‐Gal‐positive siPURPL-transfected cells compared to ctrl NHDFs. Percentage of (SA)‐β‐Gal‐positive siPURPL-transfected cells compared to ctrl NHDFs. **p* < 0.05 vs. ctrl NHDFs. (d) Western blotting analysis of LaminB1, p21 and γH2AX in siPURPL and siCTRL NHDFs; protein bands were normalized to tubulin. Histograms report γH2AX, LaminB1 and p21 protein quantification. **p* < 0.05 vs. siCTRL NHDFs. (f) Confocal analysis of γH2AX expression in siPURPL-transfected NHDFs. Scale bar = 10 μm. (e) Cell cycle analysis in siPURPL and siCTRL NHDFs using the FlowJo software (BD Bioscience). Histograms report the percentage of cell cycle distribution in siPURPL-transfected cells compared to siCTRL NHDFs. In all the graphs, bars are the mean (± *SD*) of three experiments.
